# Circulating and Disseminated Tumor Cells in the Management of Advanced Prostate Cancer

**DOI:** 10.1155/2012/135281

**Published:** 2011-08-21

**Authors:** Stephan Kruck, Georgios Gakis, Arnulf Stenzl

**Affiliations:** Department of Urology, Eberhard-Karls University, Hoppe Seyler-Straße 3, 72076 TÜbingen, Germany

## Abstract

Management of prostate cancer is recognized as one of the most important medical problems. Latest findings concerning the role of circulating (CTC) and disseminated tumor cells (DTC) have provided new insights into the biology of metastasis with important implications for the clinical management of prostate cancer patients. Most of the established methods of circulating/disseminated tumor cell enrichment use density-gradient centrifugation and immunomagnetic procedures. Reverse transcriptase polymerase chain reaction is another used detection technique. Novel methods, the CTC-chip and the epithelial immunospot assay already showed promising results. For localized and metastatic prostate cancer, significant correlations between spreading tumor cells and well-established indicators of disease activity have been demonstrated. Careful randomized prospective trials will be required to justify the routine use of CTCs/DTCs for therapy decision making.

## 1. Introduction

While significant therapeutic advances have been made for primary curative procedures, there still remains a significant risk of prostate cancer recurrence after therapy. Between 27% and 53% of all patients will develop local or distant recurrences within 10 years of initial curative-intended therapy. In addition, 16%–35% of patients will receive second-line treatment within 5 years of initial therapy [1]. Despite recent advances in treatment of advanced prostate cancer, the majority of patients with metastatic disease will die. In many cases, effective treatment of metastatic disease is delayed due to diagnostic failure of early detection by standard clinical or radiographic evaluation [2]. The presence of disseminated tumor cells at the time of primary diagnosis is assumed to be an important determinant for subsequent successful treatment and has been examined for a variety of human malignancies [3, 4]. However, the process of metastatic spread from the primary tumor site into distal organs is still not well understood. Recent studies suggest an early spread of tumor cells to lymph nodes or bone marrow (BM) referred to as “disseminated tumor cells” (DTCs) or as “circulating tumor cells” (CTCs) when present in the peripheral blood (PB) [5, 6]. Even after complete removal of the primary tumor, DTCs/CTCs can cause later development of distant metastases. There is growing evidence that spreading tumor cells indicate metastatic disease and poor prognosis [7, 8]. Although the first report on circulating tumor cells had been published in 1869 by Asworth, the lack of technology precluded further investigations on their clinical use until recently [9]. Today, technological advances in immunological and quantitative real-time PCR-based analysis enable clinicians to detect, enumerate, and characterize DTCs and CTCs in cancer patients. The monitoring of CTCs and DTCs has the potential to not only improve therapeutic management at an early stage, but also to identify patients with increased risk of tumor progression or recurrence before the onset of clinically evident metastasis. In addition, the molecular characterization of CTCs and DTCs can provide new insights into prostate cancer biology and systemic treatment in neoadjuvant or adjuvant settings.

## 2. Detection Methods of CTCs/DTCs in Urogenital Cancer

Unfortunately, the clinical applicability of most CTC/DTC detection techniques is restricted due to their high complexity, methodological limitations, and lack of standardization. DTCs and CTCs can be found in the peripheral blood and bone marrow samples of cancer patients [10]. The rare presence of CTCs in the blood system (estimated as one tumor cell per billion normal blood cells) requires the use of advanced bioengineering tools for tumor cell identification and enumeration. The sampling from the BM is more invasive and is difficult to implement in daily clinical practice. Current methods of CTC detection in the PB are limited because of comparatively lower CTC concentration rates [11, 12]. Several methods of CTC/DTC enrichment have been investigated to increase the sensitivity [7]. The most established tumor cell enrichment methods for BM and PB samples use density-gradient centrifugation and immunomagnetic procedures [13]. Cell density-based enrichment methods are based on the principle of differential cell migration according to their buoyant density. In contrast, antibody-related techniques use specific antigen patterns on the tumor cell surface to separate tumor and blood cells. For example, tumor cell-specific cell adhesion protein EpCAM is overexpressed in many types of cancer. Many approaches with EpCAM antibodies conjugated with magnetic particles followed by separation in a magnetic field have been described in literature [14, 15]. After tumor cell enrichment immunological and PCR-based molecular assays are used for the detection of CTCs/DTCs. Some methods use only detection assays without further enrichment steps to minimize tumor cell loss due to limited cell separation. Immunological approaches are based on either specific epithelial (i.e., EpCAM) or organ-specific antigens (i.e., PSA) which are exclusively expressed on tumor cells and can be analyzed by simplified automated scanning devices. Automated digital ultraspeed microscopy and the use of laser scanning have opened up new opportunities for immunocytochemical approaches in this field [16]. Due to the aforementioned methods being highly labor and time consuming, a semiautomated immunomagnetic system (CellSearch) was developed to combine both detection and enrichment of tumor cells. This technique uses ferro fluids coupled to EpCAM antibodies followed by cytokeratin staining and separation. CellSearch has already been introduced into clinical trials to monitor CTC in patients treated with new targeted therapies and was approved by the US Food and Drug Inspection Agency [17]. In addition, this measuring method has been effectively used to evaluate prognostic influence on cancer progression in various types of human cancers [18–20]. However, EpCAM-based enrichment is limited by the wide variety of protein expression levels in different tumor types [16]. Reverse transcriptase polymerase chain reaction (RT-PCR) is one of the most commonly used techniques for CTC detection and has been successfully applied in many cancer types. 

This method can detect tumor-associated molecular markers with a higher sensitivity than protein-based assays [21–23]. CTC detection by PCR depends on gene-specific oligonucleotide primers and has already been used to detect prostate cancer cells in the PB [24]. However, PCR has low specificity due to the lack of cancer-specific molecular targets in the overall majority of urogenital malignancies. In addition, tumor-associated proteins like prostate-specific antigen (PSA) are also expressed in normal cells. Therefore, PCR is extremely susceptible to errors in quantification and needs high methodical standardization, which limits its wide clinical use. A new detection method, the so called “CTC chip,” can separate tumor cells from whole blood by EpCAM-antibody-coated microspots. After this first step, CTCs are stained with antibodies against cytokeratin or tissue-specific markers, such as PSA in prostate cancer, and are visualized by automated scanning. The CTC chip demonstrated high sensitivity and specificity with doubled detection efficiency in comparison to currently available technologies such as CellSearch [25]. In patients with metastatic prostate cancer, the CTC chip identified 16 to 292 CTCs per milliliter. The CTC chip enables cell separation without harming the cellular integrity of tumor cells and allows subsequent molecular analysis. Another novel antibody-based technique is the epithelial immunospot (EPISPOT) assay, an adaptation of the enzyme-linked immunospot assay. This test is highly sensitive and detects only viable protein-excreting cells. Detection of CTCs/DTCs has shown promising results in kidney and bladder cancer patients with advanced disease. In various studies, CTCs have been detected in renal cell carcinoma patients in the PB by immunocytochemistry and PCR. Immunocytochemical methods have reached CTC detection rates between 32% and 53% in patients with metastatic disease [10, 26–28]. For PCR-based CTC analyses in renal cell carcinoma, the detection rates range between 37.5% and 49% [29–33]. In addition, Buchner et al. studied 256 BM samples of nonmetastatic RCC patients and reported a detection rate of DTCs in 25% but without any prognostic relevance [34]. The prognostic relevance of cytokeratin-positive BM DTCs was demonstrated in 55 patients with metastatic RCC compared with 256 patients without systemic involvement with significantly more DTCs being detected in metastatic disease (42% versus 25%, resp.). Multivariate analysis revealed that the presence of more than 3 DTCs was an independent prognostic factor [35]. Bluemke et al. evaluated the presence of CTCs in 233 PB samples from 154 patients with RCC after magnetic cell sorting followed by immunocytochemical staining against cytokeratin. In preliminary studies, the authors established a CD45 depletion protocol and identified 29%–42% of CTCs in patients with RCC. The frequency of cytokeratin-positive CTCs and “tumor-like” blue-stained cells without cytokeratin expression showed a significant correlation to lymph node status and presence of synchronous metastases in RCC. 

CTCs were identified in 41% of the samples, corresponding to 53% of patients. In multivariate analysis, cytokeratin-stained CTCs were identified as an independent prognostic factor for a reduced overall survival. Interestingly, postoperative blood samples (40% of sample size) showed a higher rate of CTCs compared to preoperative ones indicating increased tumor cells dissemination during surgery [27]. The role of CTCs has also been studied in patients with urothelial cell carcinoma. Urothelial cancer cells express the cell surface molecule EpCAM and can therefore be detected by iron particles coated with anti-EpCAM antibodies. Naoe et al. demonstrated that the sensitivity of the CellSearch assay was approximately 79% for the detection of CTCs derived from established urothelial cancer cell lines and mixed with peripheral blood mononuclear cells. In this study, in contrast to patients with localized disease, CTCs were only present in patients with metastatic disease [36]. CTCs might therefore represent an important tool to monitor the efficacy of chemotherapy in metastatic disease or addressing the risk of undiscovered micrometastatic disease when considering adjuvant chemotherapy for patients with locally advanced bladder cancer. In this respect, the feasibility of using the CellSearch system was recently demonstrated in patients with locally advanced nonmetastatic or metastatic disease. Moreover, the presence of CTCs in these patients was associated with a significantly reduced progression-free and cancer-specific survival [37]. Recently, Gradilone et al. showed that the bladder cancer cell marker survivin was expressed in 92% of patient with high-grade pT1 bladder cancer and the presence of survivin-expressing CTCs was an independent predictor for decreased disease-free survival [38]. 

## 3. CTCs and DTCs and Their Relevance in Prostate Cancer

Until now, prostate-specific antigen (PSA) has been the most investigated biomarker across all prostate cancer disease stages. However, in many cases, PSA is inadequate to document status of metastasis and risk of progression. The high concentration of CTCs/DTCs reported in prostate cancer patients provides novel opportunities for alternative therapeutic approaches and monitoring concepts [39]. In addition, recent data have shown that CTCs can be found at high frequency in metastatic disease underlining their potential use as surrogate markers to predict clinical outcomes and survival [17, 40–46]. Chen et al. correlated CTCs in 84 advanced prostate cancer patients with PSA, prostate-specific membrane antigen expression, and clinical parameters. Beside a high rate of intact CTCs, the authors found significant correlations between CTCs and established disease indicators (i.e., PSA), but no significant correlation for Gleason score or the type of therapy and metastasis [46]. In 2007, Shaffer et al. isolated and analyzed CTCs from PB samples of patients with advanced prostate cancer and showed that 65% of patients had five or more CTCs per 7.5 mL PB with an average CTC count of 16. In this study, CTCs were available for further epidermal growth factor receptor (EGFR) expression, chromosome ploidy, and androgen receptor (AR) gene amplification analyses [45]. The role of CTC baseline threshold value critical for survival was evaluated after immunomagnetic separation in 120 patients with progressive castration-resistant prostate cancer. Higher CTC numbers were identified predominantly in patients with bone compared to patients with soft tissue metastases and with prior chemotherapy. In univariate analysis, baseline CTCs and PSA were associated with survival without showing a threshold value [41]. Nagrath and coworkers established a microfluidic platform, the so-called CTC-chip for CTC detection and demonstrated a sensitivity rate of more than 99%. In early-stage prostate cancer, CTCs were isolated in all seven patients [25]. In a large trial leading to the FDA approval of the CellSearch system for therapeutic monitoring of castration-resistant prostate cancer, 231 patients were stratified in two prognostic groups according to CTC count (<5 or ≥5 per 7.5 mL of PB). During a follow-up period of up to 36 months under mainly docetaxel-based chemotherapies, CTC based prediction of overall survival was better than with PSA [17]. A recent study examined potential tumor-specific aberrations in the blood of cancer patients and their use as surrogate markers for the presence of CTCs. Samples were analyzed in 81 prostate cancer patients by immunospot assay and PCR-based fluorescence microsatellite analysis. Authors showed correlations between DNA plasma levels and the differentiation between tumor stage, localized and systemic disease. Moreover, CTCs correlated with tumor stage and higher Gleason scores [47]. Recently, Scher and colleagues showed a strong association between survival and CTC changes after systemic therapy in metastatic castration-resistant prostate cancer patients receiving first-line treatment [44]. 

However, the low rate of CTC in the prechemotherapy setting limits the use of CTCs as a potential biomarker for prostate cancer detection. Multiple ongoing phase III studies aim to clarify the role of CTC changes and their potential to replace established biomarkers in the monitoring of chemotherapy treated-prostate cancer patients.

## 4. Conclusion

Advanced technologies offer nowadays the opportunity to detect tumor cells in the peripheral blood and bone marrow and to estimate the risk of progression after intended curative surgery in prostate cancer patients. Despite the relatively scarce data available, DTCs/CTCs have the potential to specifically address currently controversial issues in the management of advanced prostate cancer. However, before detection of CTCs/DTCs becomes the standard of care, there is a need for further prospective randomized studies.

## References

[1] Heidenreich A, Aus G, Bolla M (2009). EAU guidelines on prostate cancer. *Actas Urologicas Espanolas*.

[2] Okajima E, Kotake T (1994). Management of advanced urogenital cancer: treatment of metastasis. *Hinyokika Kiyo*.

[3] Lugo TG, Braun S, Cote RJ, Pantel K, Rusch V (2003). Detection and measurement of occult disease for the prognosis of solid tumors. *Journal of Clinical Oncology*.

[4] Funke I, Schraut W (1998). Meta-analyses of studies on bone marrow micrometastases: an independent prognostic impact remains to be substantiated. *Journal of Clinical Oncology*.

[5] Pantel K, Brakenhoff RH (2004). Dissecting the metastatic cascade. *Nature Reviews Cancer*.

[6] Hüsemann Y, Geigl JB, Schubert F (2008). Systemic spread is an early step in breast cancer. *Cancer Cell*.

[7] Paterlini-Brechot P, Benali NL (2007). Circulating tumor cells (CTC) detection: clinical impact and future directions. *Cancer Letters*.

[8] Pantel K, Brakenhoff RH, Brandt B (2008). Detection, clinical relevance and specific biological properties of disseminating tumour cells. *Nature Reviews Cancer*.

[9] Asworth TR (1869). A case of cancer in which cells similar to those in tumors were seen in the blood after death. *Australasian Medical Journal*.

[10] Bilkenroth U, Taubert H, Riemann D, Rebmann U, Heynemann H, Meye A (2001). Detection and enrichment of disseminated renal carcinoma cells from peripheral blood by immunomagnetic cell separation. *International Journal of Cancer*.

[11] Masuda TA, Kataoka A, Ohno S (2005). Detection of occult cancer cells in peripheral blood and bone marrow by quantitative RT-PCR assay for cytokeratin-7 in breast cancer patients. *International Journal of Oncology*.

[12] Wiedswang G, Borgen E, Schirmer C (2006). Comparison of the clinical signiicance of occult tumor cells in blood and bone marrow in breast cancer. *International Journal of Cancer*.

[13] Rosenberg R, Gertler R, Friederichs J (2002). Comparison of two density gradient centrifugation systems for the enrichment of disseminated tumor cells in blood. *Cytometry*.

[14] Trzpis M, McLaughlin PMJ, De Leij LMFH, Harmsen MC (2007). Epithelial cell adhesion molecule: more than a carcinoma marker and adhesion molecule. *American Journal of Pathology*.

[15] Wu CH, Lin SR, Hsieh JS (2006). Molecular detection of disseminated tumor cells in the peripheral blood of patients with gastric cancer: evaluation of their prognostic significance. *Disease Markers*.

[16] Gerges N, Rak J, Jabado N (2010). New technologies for the detection of circulating tumour cells. *British Medical Bulletin*.

[17] de Bono JS, Scher HI, Montgomery RB (2008). Circulating tumor cells predict survival benefit from treatment in metastatic castration-resistant prostate cancer. *Clinical Cancer Research*.

[18] Cristofanilli M, Broglio KR, Guarneri V (2007). Circulating tumor cells in metastatic breast cancer: biologic staging beyond tumor burden. *Clinical Breast Cancer*.

[19] Naoe M, Ogawa Y, Takeshita K, Iwamoto S, Miyazaki A (2008). Use of the CellSearch circulating tumor cell test for monitoring urothelial cancer: two case reports of metastatic urothelial cancer. *Southern Medical Journal*.

[20] Wind J, Tuynman JB, Tibbe AGJ (2009). Circulating tumour cells during laparoscopic and open surgery for primary colonic cancer in portal and peripheral blood. *European Journal of Surgical Oncology*.

[21] Ghossein RA, Scher HI, Gerald WL (1995). Detection of circulating tumor cells in patients with localized and metastatic prostatic carcinoma: clinical implications. *Journal of Clinical Oncology*.

[22] Seiden MV, Kantoff PW, Krithivas K (1994). Detection of circulating tumor cells in men with localized prostate cancer. *Journal of Clinical Oncology*.

[23] Bossolasco P, Ricci C, Farina G (2002). Detection of micrometastatic cells in breast cancer by RT-PCR for the mammaglobin gene. *Cancer Detection and Prevention*.

[24] Helo P, Cronin AM, Danila DC (2009). Circulating prostate tumor cells detected by reverse transcription-PCR in men with localized or castration-refractory prostate cancer: concordance with CellSearch assay and association with bone metastases and with survival. *Clinical Chemistry*.

[25] Nagrath S, Sequist LV, Maheswaran S (2007). Isolation of rare circulating tumour cells in cancer patients by microchip technology. *Nature*.

[26] Meye A, Bilkenroth U, Schmidt U (2002). Isolation and enrichment of urologic tumor cells in blood samples by a semi-automated CD45 depletion autoMACS protocol. *International journal of oncology*.

[27] Bluemke K, Bilkenroth U, Meye A (2009). Detection of circulating tumor cells in peripheral blood of patients with renal cell carcinoma correlates with prognosis. *Cancer Epidemiology Biomarkers and Prevention*.

[28] Blümke K, Bilkenroth U, Schmidt U (2005). Detection of circulating tumor cells from renal carcinoma patients: experiences of a two-center study. *Oncology Reports*.

[29] Hioki T, Sugimura Y (1999). Detection of circulating cancer cells by nested reverse transcription-polymerase chain reaction of cytokeratin-19 in patients with renal cell carcinoma. *Hinyokika Kiyo*.

[30] McKiernan JM, Buttyan R, Bander NH (1999). The detection of renal carcinoma cells in the peripheral blood with an enhanced reverse transcriptase-polymerase chain reaction assay for MN/CA9. *Cancer*.

[31] Shimazui T, Yoshikawa K, Uemura H (2003). Detection of cadherin-6 mRNA by nested RT-PCR as a potential marker for circulating cancer cells in renal cell carcinoma. *International Journal of Oncology*.

[32] Ashida S, Okuda H, Chikazawa M (2000). Detection of circulating cancer cells with von Hippel-Lindau gene mutation in peripheral blood of patients with renal cell carcinoma. *Clinical Cancer Research*.

[33] Li G, Passebosc-Faure K, Gentil-Perret A, Lambert C, Genin C, Tostain J (2005). Cadherin-6 gene expression in conventional renal cell carcinoma: a useful marker to detect circulating tumor cells. *Anticancer Research*.

[34] Buchner A, Riesenberg R, Kotter I, Crispin A, Hofstetter A, Oberneder R (2003). Detection and prognostic value of cytokeratin positive tumor cells in bone marrow of patients with renal cell carcinoma. *Journal of Urology*.

[35] Buchner A, Riesenberg R, Kotter I, Hofstetter A, Stief C, Oberneder R (2006). Frequency and prognostic relevance of disseminated tumor cells in bone marrow of patients with metastatic renal cell carcinoma. *Cancer*.

[36] Naoe M, Ogawa Y, Morita J (2007). Detection of circulating urothelial cancer cells in the blood using the CellSearch system. *Cancer*.

[37] Rink M, Chun FKH, Minner S (2011). Detection of circulating tumour cells in peripheral blood of patients with advanced non-metastatic bladder cancer. *BJU International*.

[38] Gradilone A, Petracca A, Nicolazzo C (2010). Prognostic significance of survivin-expressing circulating tumour cells in T1G3 bladder cancer. *BJU International*.

[39] Allard WJ, Matera J, Miller MC (2004). Tumor cells circulate in the peripheral blood of all major carcinomas but not in healthy subjects or patients with nonmalignant diseases. *Clinical Cancer Research*.

[40] Moreno JG, Miller MC, Gross S, Allard WJ, Gomella LG, Terstappen LWMM (2005). Circulating tumor cells predict survival in patients with metastatic prostate cancer. *Urology*.

[41] Danila DC, Heller G, Gignac GA (2007). Circulating tumor cell number and prognosis in progressive castration-resistant prostate cancer. *Clinical Cancer Research*.

[42] Halabi S, Vogelzang NJ, Kornblith AB (2008). Pain predicts overall survival in men with metastatic castration-refractory prostate cancer. *Journal of Clinical Oncology*.

[43] Ramiah V, George DJ, Armstrong AJ (2008). Clinical endpoints for drug development in prostate cancer. *Current Opinion in Urology*.

[44] Scher HI, Jia X, de Bono JS (2009). Circulating tumour cells as prognostic markers in progressive, castration-resistant prostate cancer: a reanalysis of IMMC38 trial data. *The Lancet Oncology*.

[45] Shaffer DR, Leversha MA, Danila DC (2007). Circulating tumor cell analysis in patients with progressive castration-resistant prostate cancer. *Clinical Cancer Research*.

[46] Chen BT, Loberg RD, Neeley CK (2005). Preliminary study of immunomagnetic quantification of circulating tumor cells in patients with advanced disease. *Urology*.

[47] Schwarzenbach H, Alix-Panabières C, Müller I (2009). Cell-free tumor DNA in blood plasma as a marker for circulating tumor cells in prostate cancer. *Clinical Cancer Research*.

